# Identification of Relationships Between Patients Through Elements in a Data Warehouse Using the Familial, Associational, and Incidental Relationship (FAIR) Initiative: A Pilot Study

**DOI:** 10.2196/medinform.3738

**Published:** 2015-02-13

**Authors:** Thomas M English, Rebecca L Kinney, Michael J Davis, Ariana Kamberi, Wayne Chan, Rajani S Sadasivam, Thomas K Houston

**Affiliations:** ^1^The University of Massachusetts Medical SchoolDivision of Health Informatics & Implementation ScienceWorcester, MAUnited States; ^2^The eHealth Quality Enhancement Research InitiativeBedford VAMCBedford, MAUnited States

**Keywords:** Informatics for Integrating Biology and the Bedside (i2b2), data warehouse, familial relationship

## Abstract

**Background:**

Over the last several years there has been widespread development of medical data warehouses. Current data warehouses focus on individual cases, but lack the ability to identify family members that could be used for dyadic or familial research. Currently, the patient’s family history in the medical record is the only documentation we have to understand the health status and social habits of their family members. Identifying familial linkages in a phenotypic data warehouse can be valuable in cohort identification and in beginning to understand the interactions of diseases among families.

**Objective:**

The goal of the Familial, Associational, & Incidental Relationships (FAIR) initiative is to identify an index set of patients’ relationships through elements in a data warehouse.

**Methods:**

Using a test set of 500 children, we measured the sensitivity and specificity of available linkage algorithm identifiers (eg, insurance identification numbers and phone numbers) and validated this tool/algorithm through a manual chart audit.

**Results:**

Of all the children, 52.4% (262/500) were male, and the mean age of the cohort was 8 years old (SD 5). Of the children, 51.6% (258/500) were identified as white in race. The identifiers used for FAIR were available for the majority of patients: insurance number (483/500, 96.6%), phone number (500/500, 100%), and address (497/500, 99.4%). When utilizing the FAIR tool and various combinations of identifiers, sensitivity ranged from 15.5% (62/401) to 83.8% (336/401), and specificity from 72% (71/99) to 100% (99/99). The preferred method was matching patients using insurance or phone number, which had a sensitivity of 72.1% (289/401) and a specificity of 94% (93/99). Using the Informatics for Integrating Biology and the Bedside (i2b2) warehouse infrastructure, we have now developed a Web app that facilitates FAIR for any index population.

**Conclusions:**

FAIR is a valuable research and clinical resource that extends the capabilities of existing data warehouses and lays the groundwork for family-based research. FAIR will expedite studies that would otherwise require registry or manual chart abstraction data sources.

## Introduction

### Overview

Over the last several years there has been widespread development of medical data warehouses. The National Institutes of Health (NIH) funded the development and adoption of Informatics for Integrating Biology and the Bedside (i2b2). The i2b2 scalable informatics framework enables researchers to use existing clinical data for discovery research that may be combined with genomic data. This framework can be extended for new and unanticipated data types, as well as for functionality [1-3]. Unlike disease registries, the i2b2 architecture is not limited by project-specific designs, implementation, and policies for data use. Thus, the potential to extract high-quality data for cohort identification, surveillance, and predictive clinical tools has never been greater [4].

Current data warehouses focus on individual cases, but lack the ability to identify family members who could be of interest for dyadic or familial research. Currently, the patient’s family history in the medical record is the only documentation that we have to understand the health status and social habits of family members. Recent projects have attempted to gain a better view of family history using natural language processing, but these studies have not linked individual records to each other [5]. Recent literature has demonstrated the capability of linking children with their parents through electronic health records (EHRs) using guarantor and emergency contact information [6].

Many research projects have an interest in families or multiple members of the same familial unit, but study recruitment can often be difficult due to the clinical system’s lack of efficiency in documenting biological ties. Consider the clinical research informatics study described below and the benefit of using the Familial, Associational, & Incidental Relationships (FAIR) tool, which is integrated in the clinical data warehouse.

### Case Study Using the FAIR Initiative

A research project required the identification of a cohort of children with autism. The research team was able to identify a cohort of children using diagnostic codes in the clinical data warehouse. In the warehouse, there was also an identifier for children who had a specimen in the institutional biorepository. However, the project required information about the autistic children’s mothers. The investigators sought consultation with the leaders of the biomedical informatics component of their institution’s NIH-funded Clinical and Translational Sciences Award. The challenge was to use the data warehouse to identify how many mothers of the identified cohort of children had also received care in the clinical system. Once identified, both phenotypic and biorepository data would be available on this cohort of mothers for the study.

Finding the mothers of children is often not possible because family members are not linked in the underlying databases. FAIR could aid researchers in the identification of eligible children and also identify which individuals have a mother with a biospecimen stored in our biorepository.

### Study Overview and Goal

In response to this research challenge, this paper describes the methodology we developed, the preliminary testing of the method, and the implementation of FAIR, based within the i2b2 framework, for linking data on children and their mothers. We focused our initial testing on the capability of FAIR to accurately find mothers of a randomly selected group of children. The FAIR initiative i2b2 plugin will identify and extract data into cohorts and aid in the understanding of disease by examining the demographic, clinical, and genetic variations of risk through families and generational cohorts. The goal of this study is to develop an algorithm that uses data commonly available in EHRs to find mothers of a group of 500 children. We hope that this algorithm will be able to link children to their mothers accurately enough to enable recruitment for future research studies.

## Methods

### Study Design

The FAIR initiative had two phases. In Phase I, we conducted a manual chart abstraction to evaluate the test characteristics (eg, sensitivity, specificity) of potential linkage variables selected to link an index population of 500 children and their mothers. In Phase 2, we developed an i2b2 Web app designed to construct linkages and present the results through the Web interface. FAIR was reviewed and approved by the University of Massachusetts Medical School Institutional Review Board.

### Phase I: Evaluating the Test Characteristics of Potential Linkage Variables

In Phase I of FAIR, we measured the test characteristics of three potential linkage variables in a sample of 500 children.

#### Setting and Sample

We included patients (children and their mothers) who had received care in the University of Massachusetts Memorial Health Care (UMMHC) system. We limited the population to patients in the 774 area code, which covers a large portion of the catchment area for the UMMHC system. The initial index population consisted of 13,090 children who were 17 years of age or younger. We randomly selected 500 of those children as our test cohort. For this pilot study, we decided to focus exclusively on finding mothers, versus fathers, because the maternal-child relationship was the focus of several of our colleagues’ research, including the investigation of medications used during pregnancy and possible outcomes for the child.

#### Data Elements

We identified a set of three variables that could potentially link children and their mothers: insurance number, phone number, and address. These variables were chosen because they are available for the majority of patients in our data warehouse and have face validity to support linkage between child and mother. While we considered other identification variables, such as name and race, this data is not often specific to single or small groups of families, therefore, this data was excluded as part of this query.

#### The Comparison Reference Standard: Manual Chart Abstraction

In order to evaluate the accuracy of our automated match process, we conducted a manual chart audit to validate the findings. During the manual chart audit, we were able to discretely explore numerous documents and data points that were not available for extraction. We started the process by reviewing the child’s chart to look for references to other people who might be related. We used search functions native to our EHR to determine if individuals had the same phone or insurance number and checked those charts to find a match, similar to what was done with the automated match process. In addition, we reviewed documents that were scanned into charts to look for relatives. In many cases, we found that signed consent forms in the child’s chart listed a parent’s name and their relationship. We also found more detailed insurance information, including guarantor, which often led to mothers who were not found using phone or insurance number.

#### The Familial, Associational, & Incidental Relationships Automated Linkage Algorithm

The automated linkage algorithm occurred in two steps. The first step was to find patients that had the same insurance number, phone number, or address as a child in our study cohort. In the second step, once the linkages were made, the system was designed to differentiate the relationship. For this study, we designed the algorithm to identify potential child-mother linkages only. In cases where multiple automated linkages were identified for a single child, the system selected the oldest linked female to enhance the linkage accuracy and avoid sibling identification. If that person was 15 to 50 years older than the child, the system classified the relationship as a child-mother link.

#### Analyses

We compared the use of the individual identifiers, as well as identifier combinations, to the reference standard results to determine the sensitivity (ie, finding mothers that are in the system) and specificity (ie, not finding mothers that are not in the system) of the process. This helped determine the positive predictive value (PPV) and negative predictive value (NPV) of the FAIR automated linkage algorithm. In addition, we reviewed the false positive and false negative linkages and compared them to true positive linkages to identify characteristics of the child that might modify the success of the automated linkage algorithm. It is important to note that NPV can be misleading because our manual linkage method allows for the possibility of missing some mothers who do exist in the database.

### Phase II: Developing and Testing an i2b2 Web App

In Phase II, we developed a set of i2b2 plugins that can be used to view the diagnoses of a patient’s relatives. The “FAIR-correlated” patients are available through the Massachusetts Integrated Clinical Academic Research Database (MICARD), the University of Massachusetts’ implementation of the i2b2 informatics platform for clinical research.

This software was built first in our development server and then integrated in the production environment. We tested the system in a number of ways to make sure that the software worked as intended. Software inspection (SI) was used to find errors, omissions, and anomalies in the source code. The SI was conducted using a peer-review process of developers in our academic division along with the primary developer for this study. We also conducted unit testing to verify whether independent units of code were working correctly [7]. Moreover, integration testing was used to verify the accuracy of the software [8]. Mock test cases with input and predicted output datasets were then developed to conduct the unit and integration testing.

## Results

### Overview

The average age of the children in this study sample was 8 years of age (SD 5). The cohort was 52.4% (262/500) male. Of all the children, 51.6% (258/500) were identified as white in race (see Table 1)**.**

**Table 1:** Characteristics of index patients (children) in the test sample (n=500).

Patient characteristic		n (%)
**Age**		
	Less than 1 year	12 (2.4)
	1 to 5 years	180 (36.0)
	6 to 10 years	138 (27.6)
	11 to 15 years	124 (24.8)
	16 years and over	46 (9.2)
**Gender**		
	Male	262 (52.4)
	Female	238 (47.6)
**Race/ethnicity**		
	White	258 (51.6)
	African American	39 (7.8)
	Hispanic	71 (14.2)
	Other	66 (13.2)
	Unknown	66 (13.2)

### Phase I: Evaluating the Test Characteristics of Potential Linkage Variables

#### Overview

The identifiers used for FAIR were available for the majority of patients: insurance number (483/500, 96.6%), phone number (500/500, 100%), and address (497/500, 99.4%). Using the manual review process, we determined that the prevalence of having an identifiable mother in the system was 80.2% (401/500). Initially, we considered each identifier alone. Subsequently, we used these results to also test combinations of the identifiers.

**Figure 1:**
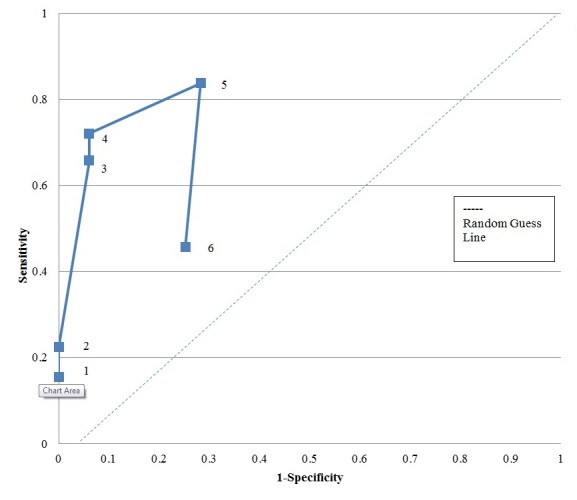
Receiver operating characteristics (ROC) curve of matching methods (1=insurance and phone, 2=insurance only, 3=phone only, 4=insurance or phone, 5=insurance, phone, or address, 6= address only).

The sensitivity and specificity for each identifier and combination is presented in Table 2. The corresponding receiver operating characteristics (ROC) curve is shown in Figure 1. Each initial identifier produced differing results for sensitivity, specificity, PPV, and NPV in this analysis.

**Table 2:** Test characteristics of individual identifiers and combinations compared with verification of child-mother linkage by manual chart abstraction.

Identifier(s)	Sensitivity (n=401), n (%)	Specificity (n=99), n (%)	Positive predictive value, n/n (%)	Negative predictive value, n/n (%)
Insurance identification number	90 (22.4)	99 (100)	90/90 (100)	99/410 (24.1)
Phone number	264 (65.8)	93 (94)	264/270 (97.8)	93/230 (40.4)
Address	182 (45.4)	74 (75)	182/207 (87.9)	74/293 (25.3)
Insurance *or* phone	289 (72.1)	93 (94)	289/295 (98.0)	93/205 (45.4)
Insurance, phone, *or* address	336 (83.8)	71 (72)	336/364 (92.3)	71/136 (52.2)
Insurance *and* phone	62 (15.5)	99 (100)	62/62 (100)	99/437 (22.7)

#### Data Elements

##### Insurance Number

Matching children to their mothers based on insurance number alone resulted in low sensitivity (90/401, 22.4%) but perfect specificity (99/99, 100%). The insurance number was the only identifier that did not contribute to any false positives with PPV (90/90, 100%). The NPV for insurance number alone was also quite low (99/410, 24.1%). A major issue was that family insurance numbers are not assigned by many companies. In particular, most safety net insurers use individual numbers. Thus, querying by insurance number alone will exclude families of lower socioeconomic status. Additionally, working with insurance numbers requires restructuring the data due to the length of the numbers, which often vary by provider. Typically, the insurance numbers contain two sets of digits: the first number specifies the account, and the second number specifies the individual. Hence, prior to matching, the digits specifying the individual need to be removed.

##### Phone Number

Unlike insurance, phone number was far more sensitive (264/401, 65.8%) in identifying true relationships with only a small drop in specificity (93/99, 94%). The PPV (264/270, 97.8%) was high, yet the NPV (92/230, 40.4%) was still quite low, though better than when only insurance was used. Since phone numbers were available for all of the patients, it was the only consistent way to find family members of patients who were uninsured or had insurance from a company that assigned unique numbers rather than family numbers.

##### Address

Address had sensitivity of 45.4% (182/401) for identifying relationships, a specificity of 75% (74/99), a PPV of 87.9% (182/207), and an NPV of 25.3% (74/293). Variation in how patients’ addresses were entered into the system was a major issue. In particular, it was common to find addresses for an apartment building that were lacking unit numbers. This inaccurate data resulted in a large number of false positives.

##### Insurance or Phone Number

The algorithm we used for the primary comparison required either insurance number or phone number. Since the primary application of FAIR is cohort discovery to enable recruiting, we placed a high value on the PPV. This method resulted in the highest combined sensitivity (289/401, 72.1%) and specificity (93/99, 94%) of all of our tests. The PPV (289/295, 98.0%) was high, but the NPV (93/205, 45.4%) was low.

##### Insurance Number, Phone Number, or Address

As anticipated, using any of the three identifiers would result in the most sensitive method (336/401, 83.8%), but with a lower specificity (71/99, 72%). It provided a high PPV (336/364, 92.3%), but a low NPV (71/136, 52.2%).

##### Insurance and Phone Numbers

Requiring both insurance and phone numbers in the query was the most restrictive combination tested, but the utility was dominated by the use of insurance alone. Requiring multiple variables to match patients limited the sensitivity (62/401, 15.5%), but the specificity (99/99, 100%) was perfect. The PPV was 100% (62/62) because matching was based partially on insurance. The NPV (99/437, 22.7%) for this method was the lowest of all the matching methods tested.

Based on the performance of the individual variables, we decided to drop the address identifier from our final algorithm and use only phone number or insurance number. Once initial matches were made, we had to differentiate the relationships of the patients and determine the one to be selected if multiple potential mothers were found. We limited the group to females who were 15 to 50 years of age older than the child in the cohort. If we found multiple matches, we considered phone and insurance numbers first, then insurance number, and then phone number. If, at that point, we still had multiple matches, we chose the oldest female from the group.

The algorithm is run outside of i2b2 and loaded into the schema. This allows institutions to use whatever matching algorithm works best. Flexibility is crucial since the data available for matching differs by institution.

#### The Comparison Reference Standard Versus the Automated Linkage Algorithm

In our comparison of the manual and FAIR automated linkage study, we uncovered some situations where FAIR was less successful. One challenge was the inability to differentiate biological and nonbiological parent cases. A second set of challenges was related to socioeconomic status.

The system is not able to differentiate between parents and stepparents. In some instances, we found that the stepparent had more variables in common with a child than a biological parent. Thus, any use of FAIR will require an additional manual review to confirm the biological connection of the identified relationship.

Unfortunately, populations of lower socioeconomic class had a lower chance of matching for several reasons. First, most safety net insurance plans (eg, Medicaid) have unique numbers for individuals, but do not use family numbers. This is an important linkage variable because, although specific, it was not sensitive as it missed all patients with these insurance plans. In addition, if a family frequently changed phone numbers, the chances of making a positive match decreased. Lower income households are more likely to have unstable phone service, potentially changing plans frequently as finances require, thus making it difficult to identify a familial linkage based on phone number. Finally, we found that individuals of lower socioeconomic class often resided in multi-unit dwellings, so these locations would have the same street address. Because address data often lacked unit numbers, false positives were identified in the lower socioeconomic strata, reducing the specificity for this important variable.

### Phase II: Developing and Testing an i2b2 Web App

As part of this initiative, a set of i2b2 Web Client plugins were engineered to enable researchers to utilize relationship information in their studies. The plugin displays the FAIR members of each patient in an i2b2 patient set, and then allows the user to select the relevant groups of patients and their corresponding FAIR members on which to tabulate their concepts. In other words, it enables a researcher to quickly trace the occurrences of certain concepts in various FAIR groups.

The i2b2 Web Client is a Web-based interface to the i2b2 Hive, which enables a user to access the i2b2 data through a Web browser. The primary advantage of the Web Client is that the software does not have to be specifically installed on the user’s computer or device. In addition, because the Web Client is Web browser based, researchers using non-Windows computers or devices (eg, tablets) may also access the i2b2 data, which aids greatly in the enterprise-wide rollout of the i2b2 across an institution [9].

The architecture of the i2b2 Web Client also facilitates the addition of plugins supporting new, specific analytic functions. The FAIR Concept Tracer is such a plugin that facilitates the access of any available FAIR data for rendering. In general, adding a new plugin to the i2b2 Web Client is rather straightforward and does not require any stoppage of an institution’s i2b2 installation. The FAIR Concept Tracer is packaged with a detailed installation guide and a user document, along with the source code [10]. So far, there have not been any problems reported by other institutions concerning the incorporation of the plugin. We estimate that all the software required for FAIR could be installed in less than half an hour.

The i2b2 framework allows the storage and access of patient data for research purposes. We introduced an additional provision to expedite the capture and storing of any such FAIR information in self-defined—by each institute—simple eXtensible Markup Language (XML) forms. A set of tools has been developed to facilitate the storing and rendering of these FAIR data.

Using our FAIR schema, the incorporation of the FAIR data does not require any schematic and/or hive-cell code changes. The FAIR database administrator toolset (DBA Toolset) was developed to enable the incorporation of the new FAIR data into the existing i2b2 database while observing such constraint. The FAIR output can be extracted into an Excel spreadsheet (XLS) or a comma-separated-values (CSV) file.

We conducted some initial FAIR usability testing with users in the i2b2 community and the feedback has been positive. Users felt that the way the data were presented, as well as the flexibility of the system to modify the FAIR data structure, was excellent. The approach and methodology will be made available to all academic institutions that are part of the i2b2 community.

**Figure 2:**
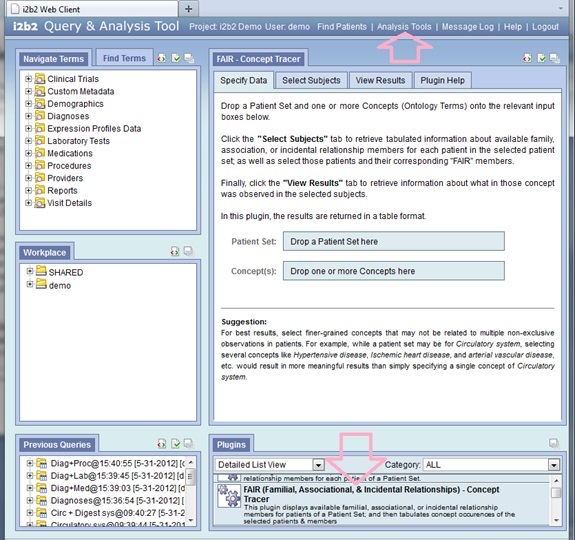
Specify Data window for the FAIR Concept Tracker.

**Figure 3:**
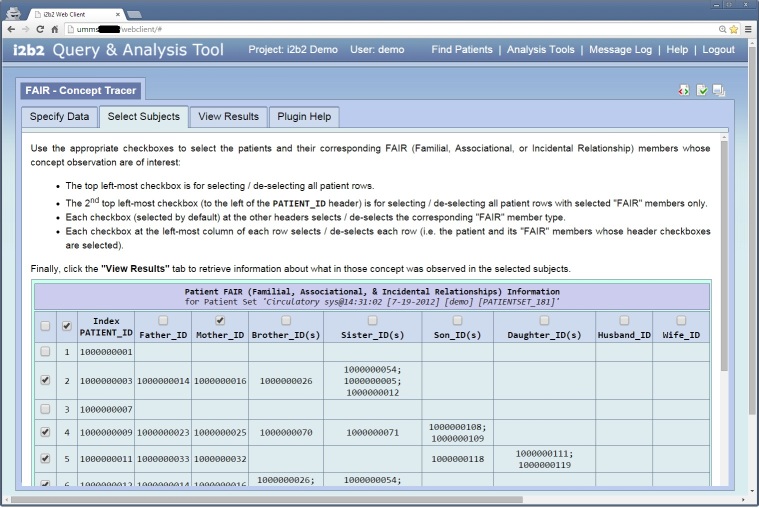
Select Subjects window for the FAIR Concept Tracker.

**Figure 4:**
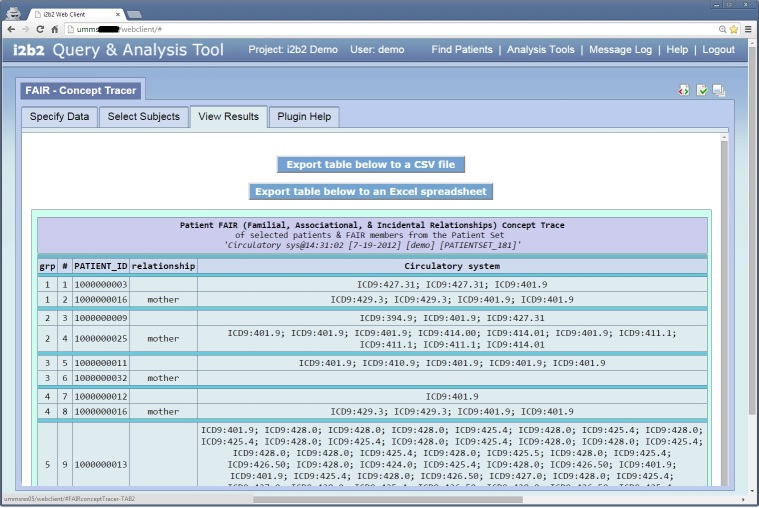
View Results window for the FAIR Concept Tracker.

Figure 2 shows the *Specify Data* window from the FAIR Concept Tracker. This window allows the user to load the desired *Patient Set*—from either the *Workplace* or the *Previous Queries* panel—and one or more *Concepts* and drop them into the appropriate drop-in boxes. Figure 3 shows the *Select Subjects* window from the FAIR Concept Tracker, which allows the user to select appropriate subjects for tracing the selected *Concepts* in the “related” individuals. Figure 4 shows the *View Results* window from the FAIR Concept Tracker. This window displays a group of patients that match, the identification number of each patient, the relationship of each group member, and the circulatory diagnoses of each patient. This example shows a view of how a fully functional system would look once all relationships, beyond that of the child and mother, have been defined.

## Discussion

### Principal Findings

The FAIR method is useful for finding potential dyadic cohorts. Identifying familial linkages in the phenotypic data warehouse can be valuable in cohort identification and in beginning to understand the interactions of diseases among families [11-13]. The optimal combination of variables was to find a match either using the insurance or phone number. However, that is assuming that sensitivity, specificity, positive predictive value, and negative predictive value are of equal importance for a given project. As noted, the automated matching algorithm was imperfect and was less successful for matching family members at lower socioeconomic levels.

In the aforementioned case study, we discussed finding mothers of autistic children. The investigators desired a tool that was able to comprehensively identify as many child-mother linkages as possible. Thus, if a linkage was not found, the case was “ruled out” as valuable for the study with some degree of certainty. The investigators wanted a highly sensitive linkage algorithm, which minimized false negatives. The most sensitive linkage was a combination of all variables with *or* logic (ie, if any of the variables matched, a linkage was identified). However, the investigators were aware that many of the potential linkages were false positives, and additional manual review would be required.

Other studies could be considered where the task is not to be comprehensive, but rather to have an algorithm that identifies only true child-parent linkages, or maximizes specificity. For these studies, the insurance identification number is the single most effective linkage variable. However, investigators must recognize that many potential linkages will be missed. Maximizing specificity has repercussions, including drastically reducing sensitivity. This is especially problematic as the insurance identification number linkage variable was much less successful in lower socioeconomic groups.

### Challenges for the Familial, Associational, & Incidental Relationships Tool

As noted, none of the linkage variables were perfect. There are certainly caveats to using data collected for clinical purposes for research [14-16]. In our validation, we found several challenges, including the inability to differentiate biological versus nonbiological parents. This problem is not likely to be solved easily with structured data elements and will likely require natural language processing of clinical notes to improve the algorithm. Also, the system was less successful with families at lower socioeconomic levels due to differences in insurance numbers, phone numbers, and street addresses.

The concept of FAIR poses new and important challenges to patient privacy in the context of clinical data being used for research purposes. FAIR facilitates linkage of information from one patient to another. Use of this identified data would require consent from both subjects to proceed. The University of Massachusetts Medical School has approved the implementation of the FAIR plugin for our de-identified i2b2 data warehouse, as the linkage is made before all identifiers that link back to patient charts are removed. However, different institutions may have different policies about relational linkage studies.

### Limitations

Our initial evaluation of FAIR was limited to a single cohort of 500 children. We did not limit our selection of children to a specific diagnosis, as some diagnoses (eg, those that vary with socioeconomic status) may have influenced the test characteristics analyses. We purposely chose the children at random, enhancing the potential generalizability across diagnoses. However, this also means that our results may not be representative of targeted disease cohorts, and future validations of FAIR in specific projects are needed. In addition, we have only tested the indicator linkages in a single clinical data warehouse at a single institution. This data warehouse is representative of one institution, therefore, this study may not be generalizable to other institutions or hospitals that house EHRs with differing data points or levels of completeness. Further experimentation through the informatics community—taking advantage of the NIH-funded Clinical and Translational Sciences Awards network and the widespread availability of the i2b2 platform—is warranted.

### Conclusions

FAIR is a valuable research and clinical resource that extends the capabilities of existing data warehouses and lays the groundwork for family-based research. FAIR will expedite studies that would otherwise require registry or manual chart abstraction data sources. Moreover, the knowledge that can be gained through biological ties is essential to the future prevention and management of complex diseases, such as asthma, depression, and specific childhood conditions. The potential for FAIR goes beyond child-parent relations. It could be used to identify geographically related cohorts of patients in a clinical data warehouse (eg, those living close to an environmental hazard or those living in an underserved area). Thus, the flexibility of FAIR should enable a wide variety of research. Our hope is that FAIR will be an innovative tool that will aid future researchers in their first steps towards predictive, personalized, and preemptive medicine.

The FAIR plugin is now available to all users of i2b2 [17]. Our matching algorithm is simple enough that it could be adapted to most clinical data warehouses with basic demographic, geographic, and insurance data available. Further research is needed to demonstrate the value of FAIR in concrete clinical research informatics projects.

## Abbreviations

CSV: comma-separated-values
DBA Toolset: database administrator toolset
EHR: electronic health record
FAIR: Familial, Associational, & Incidental Relationships
i2b2: Informatics for Integrating Biology and the Bedside
MICARD: Massachusetts Integrated Clinical Academic Research Database
NIH: National Institutes of Health
NPV: negative predictive value
PPV: positive predictive value
ROC: receiver operating characteristics
SI: software inspection
UMMHC: University of Massachusetts Memorial Health Care
XLS: Excel spreadsheet
XML: eXtensible Markup Language
